# Dipeptidyl peptidase-4 inhibitors improve arterial stiffness, blood pressure, lipid profile and inflammation parameters in patients with type 2 diabetes mellitus

**DOI:** 10.1186/s13098-016-0144-6

**Published:** 2016-03-22

**Authors:** Lea Duvnjak, Kristina Blaslov

**Affiliations:** Vuk Vrhovac Clinic for Diabetes, Endocrinology and Metabolic diseases, Merkur University hospital, Dugi dol 4a, Zagreb, Croatia; School of Medicine, University of Zagreb, Zagreb, Croatia

**Keywords:** Dipeptidyl peptidase-4, Type 2 diabetes mellitus, Blood pressure, Augumentation index

## Abstract

**Background:**

This uncontrolled open label study evaluated the effect of dipeptidyl peptidase-4 inhibitors (DPP-4i): sitagliptin and vildagliptin on augmentation index standardized for 75 beats per minute (cAiX@75), blood pressure (BP), lipid profile and high-sensitivity C-reactive protein (hsCRP) in patients with type 2 diabetes mellitus (T2DM).

**Methods:**

Fifty-one well-regulated T2DM patients were randomly assigned to either sitagliptin or vildagliptin (100 mg/day) for 3 months continuing their previous treatment. Lipid profile, cAiX@75, hsCRP, glycated hemoglobin (HbA1c) were measured at baseline at 4, 8 and 12th week were accessed. cAiX@75 and pulse wave velocity (PWV) were determined by SphygmoCor device.

**Results:**

Following DPP-4 treatment there was a significant reduction in total serum cholesterol (5.18 vs 4.62 mmol/L), low-density lipoprotein (2.89 vs 2.54 mmol/L), hsCRP (3.21 vs 1.95 mg/L), cAiX@75 (24.5 vs 22.3) and central systolic BP (131.8 vs 119.5 mmHg). The sitagliptin treated group reached cAiX@75 reduction earlier in the study period while neither sitagliptin or vildagliptin use resulted in the significant HbA1c reduction.

**Conclusion:**

The treatment with DPP-4i: sitagliptin and vildagliptin provides favorable metabolic and vascular effects beyond glucose-control. Further studies are required to elucidate their implication in metabolic pathways.

## Background

According to the Framingham study type 2 diabetes mellitus (T2DM) is associated with a twofold to fourfold increased risk for cardiovascular disease (CVD) [1]. This is due to diabetes related metabolic disorders: chronic hyperglycemia and dyslipidemia along with oxidative stress and low grade inflammation [2]. Although antihyperglycemic drugs improve glycemic control, the cardiovascular benefits to certain subgroups of T2DM patients has been established only for metformin and pioglitazone [3, 4]. There is a lack of evidence that the therapeutic strategies targeting pancreatic β cell dysfunction and insulin resistance (IR) reduce the cardiovascular risk in patients with diabetes [5]. This notion raises the need for alternative therapies that provide substantial benefits without side effects. Among emerging anti-diabetic candidates, incretin-based therapies carry a special cardiovascular implication.

Incretins are a group of gastrointestinal hormones, among which the glucagon-like peptide 1 (GLP-1) and the glucose dependent insulinotropic peptide (GIP) are released in response to nutrient ingestion, stimulating insulin and suppressing glucagon secretion [6]. GLP-1 also seems to have some beneficial effect on pancreatic β-cells preservation in experimental models [7]. However, this actions are limited due to its rapid inactivation by the dipeptidyl peptidase-4 (DPP-4) enzyme [8, 9]. DPP-4 inhibitors spare the GLP-1 breakdown and improve glycemic control by consequential increase insulin secretion [9, 10].

Beyond its beneficial action on metabolic control, GLP-1 seems to exert favorable cardiovascular effect mediated partially through a specific GLP-1 receptor on cardiomyocytes, vascular endothelium and vascular smooth muscle cells [10]. The administration of inherent GLP-1 causes acute animal aortic dilation [11]. This observed vasodilatory properties are probably mediated through GLP-1 metabolites and independently of the GLP-1 receptor, acting instead through an NO/cGMP-dependent mechanism [12].

DPP-4 has several non-incretin substrates involved in inflammation, immunity and cardiovascular system [13] and its expression on endothelial surface suggests that its inhibition might reduce the vascular tone [14]. Some clinical data suggest the beneficial effects of DPP-4 inhibitors on hypertension, dyslipidemia and CRP have been cited in the literature [15–18]. This suggests that those might have a potential to reduce the CVD burden among patients with T2DM.

Arterial stiffness (AS) is a strong independent predictor of CVD in several populations, including T2DM patients [19] being increasingly recognized as a surrogate end point for CVD [20]. The effect of DPP-4 inhibition on AS is still a matter of debate [15–17] and the direct comparison of different DPP-4 inhibitors impact on CVD risk factors is yet to be established.

Thus, we aimed to evaluate the effect of DPP-4 inhibitors on CVD risk factors: central obesity, lipidemia, hsCRP, glycaemia and blood pressure with a special emphasis on AS and related parameters, i.e. central pulse pressure (PP), central blood pressure (cBP) and pulse wave velocity (PWV) in metabolically well regulated T2DM patients. Second we compared the effect of two different DPP-4 inhibitors: sitagliptin and vildagliptin on the observed parameters.

## Methods

This was an uncontrolled open-label, parallel-arm, randomized 12 week study conducted in the In-patient Clinic for Diabetes, endocrinology and metabolic diseases Vuk Vrhovac, Zagreb, Croatia.

Type 2 diabetic patients of either gender, aged between 40 and 75 years were eligible for the inclusion in the study. Selected non-inclusion criteria were insulin or (current or previous 3 months) treatment with any incretin-based treatment strategy including GLP-1 analogues and DPP-4 inhibitors. Furthermore, patients with macrovascular complications such as significant arterial obliteration detected by imaging methods, an acute cardiovascular event (e.g. myocardial infarction), unstable angina or stroke within six months prior to enrollment, impaired glomerular filtration rate (<60 ml/min/1.73 m^2^) and urine albumin excretion rate >300 mg/24 h.

After the screening period patients were randomized to receive either sitagliptin (50 mg twice daily) or vildagliptin (50 mg twice daily) in addition to continuing their antidiabetic background treatment. Also, the antihypertensive and lipid-lowering drugs were unchanged during the study period. Randomization procedures were performed by principal investigator or his/her delegate by physical method of randomization using shuffle sealed envelopes with treatment allocations inside. Patients both groups were instructed to strictly maintain dietary habits and daily activities during the course of the study. They were assessed at the outpatient visit four times: at baseline, 4, 8 and 12th week. Blood samples for biochemical measurements were collected at each visit as well as physical examination including anthropometric parameters while the carotid-femoral (cf) PWV and 24-h blood pressure record was measured at baseline and at the end.

The study protocol was approved by the Ethic Committee of the Merkur University Hospital, Zagreb, Croatia and the study was performed according to Declaration of Helsinki and “good clinical practice” (GCP) guidelines. Written informed consent was obtained from all the study participants before the study entry.

The central aortic pressure waveform can be used to determine central systolic and diastolic BP, (dBP) central PP and augmentation pressure (AP). Central PP and augmentation index (cAIx; AP as a proportion of PP) are markers of AS showing good correlation with cardiovascular morbidity an mortality [21, 22]. The central arterial waveform was derived by using the SphygmoCor™ System (AtCor Medical, Sydney, Australia). The radial artery waveform was recore from the radial artery at the wrist, using high-fidelity applanation tonometry (Millar Instruments, Houston, Texas). The SphygmoCor™ System automatically generates the corresponding central (aortic) waveforms from an averaged radial artery waveform. From the central systolic and diastolic BP as well as AP and cAIx were derived. The cAIx was normalized to hearth rate of 75 beats per minute (cAIx@75).

PWV is a direct measure of AS of large arteries. For the determination of aortic PWV, waveforms of the common carotid artery and femoral artery were obtained using SphygmoCor™. PWV was calculated as the distance between suprasternal notch and the femoral artery recording site, and divided by the time interval between the feet of the flow waves.

Three readings were obtained from the right arm of seated participants while their arm was supported at heart level; 1 trained observer took all measures using an automatic office BP device (OMRON Intellisense HEM-907) according to JNC VIII criteria [23]. For 24-h ambulatory blood pressure (ABP) measurements, participants were fitted with a SpaceLabs 90207 ABP monitors. The cuff was secured around the participant’s non-dominant arm, and it remained there during the entire test. Measurements were automatically repeated if an error occurred. The adult cuff and the large adult cuff were used for arm circumferences of 24–31 and 32–42 cm, respectively.

Body weight (BW) was measured with light clothing on a balance scale and height to the nearest 0.5 cm using a meter that was stabilized on the wall. BMI was calculated as weight (kilograms) divided by height (meters) squared. Waist circumference was measured at minimal respiration by a flexible tape parallel to the floor and immediately above the iliac crest.

All laboratory tests were performed after a 12 h overnight fast. Biochemistry, including glucose, lipid levels (TC; high density lipoprotein cholesterol, HDL-C; and TG) liver enzymes (alanine transaminase, ALT; aspartate transaminase, AST, hsCRP and creatinine, were evaluated on an Olympus analyzer (Olympus AU600, Olympus Optical Co., Tokyo, Japan). Low-density lipoprotein cholesterol (LDL-C) was calculated using Friedewald’s formula [24].

To perform a formal sample size calculation the primary endpoint was set to be the effect of sitagliptin and vildagliptin on central SBP [15]. We estimated that a total of 51 patients will be needed for this two-treatment parallel-design study with the probability (power) of 99 percent that the study will detect a treatment difference at a two-sided 0.05 significance level. All the data are entered in triplicate in Open Document Spreadsheet database and the analysis was performed using SPSS 17.0 (Chicago, IL, USA).

Normality of distribution was confirmed by Shapiro–Wilk test prior to further analysis. Normally distributed data were compared by paired t test and two-way ANOVA and expressed as mean ± standard deviation. A two sided P value <0.05 was considered significant.

## Results

Fifty-one patient were enrolled in the study and all of them completed all four visits. There were 30 (58.8 %) males. Vildagliptin 2 × 50 mg/day was added to current diabetes therapy reigment in 20 (39.21 %) patients at the point treated with metformin only (up to 3 g; N = 17) and 3 patients with gliclazide 60 mg/day + metformin 2 g/day. Sitagliptin 2 × 50 mg/day was added to 31 (60.79 %) patient treated with metformin (up to 3 g/day; N = 30) and one patient treated with metformin (2.5 g and gliclazide 60 mg/day).

After 12-weeks of treatment with DPP-4 inhibitors, BMI remained almost unchanged [(28.8 (3.5) kg/m^2^ vs 28.6 (3.5) kg/m^2^), p = NS] while the waist circumference significantly decreased mean HbA1c value remained unchanged (Table 1). The group experienced a mean −4.41 (2.03) mmHg of 24-hour SBP reduction and −4.65 (2.36) mmHg 24-hourDBP reduction. Total and well as LDL cholesterol significantly dropped as well as hsCRP concentration. The treatment also resulted in triglycerides concentration reduction (Table 1).

**Table 1:** Clinical characteristics at the begining and at the study end (N = 51)

	Baseline	12-weeks after	P
Waist circumference (cm)	103.3 (12.3)	101.4 (11.8)	0.012
24 h SBP (mmHg)	125.74 (13.41)	123.54 (10.87)	<0.001
24 h DBP (mmHg)	74.49 (7.59)	73.67 (7.45)	0.281
HbA1c (%)	6.9 (1.1)	6.9 (0.7)	0.461
Cholesterol (mmol/L)	5.18 (1.0)	4.62 (1.60)	0.001
HDL-C (mmol/L)	1.29 (0.24)	1.31 (0.23)	0.973
LDL-C (mmol/L)	2.89 (0.84)	2.54 (0.85)	0.005
Triglycerides (mmol/L)	2.26 (1.35)	1.81 (0.91)	0.001
hsCRP (mg/L)	3.21 (0.86)	1.95 (1.59)	0.009

Data are expressed as mean (SD), *HbA1c* gycated haemoglobin, *Chol* cholesterol, *HDL*-*C* high density cholesterol, *LDL*-*C* low density cholesterol, *Tgc* triglycerides, *hsCRP* high sensitive C-reactive protein

A significant reduction in cAIX@75, central SBP, DBP as well as office SBP and DBP was observed (Table 2). The PWV change of −0.40 (0.90) m/s was also notified, however, it did not reach the statistical significance [8.59 (0.33) vs 8.42 (0.28), p = 0.816].

**Table 2:** Hemodynamic responses to DPP-4 inhibitors in patients with type 2 diabetes during treatment

N = 51	Week 0	Week 4	Week 8	Week 12	P
AiX@75	24.5 (9.3)	23.4 (10.5)	23.2 (7.9)	22.3 (6.8)	0.031
cSBP (mmHg)	131.8 (12.9)	128.6 (11.7)	124.6 (10.5)^a^	119.5 (15.4)^a^	0.001
cDBP (mmHg)	79.8 (6.8)	79.0 (8.2)	77.5 (11.0)	77.2 (14.1)	0.003
oSBP (mmHg)	141.8 (10.2)	139.1 (13.1)	135.1 (12.1)^a^	131.8 (18.3)^a^	0.042
oDBP (mmHg)	82.7 (1.9)	78.8 (12.4)	78.2 (7.6)	75.6 (10.5)	0.001

Data are expressed by mean (SD), *AIX@75* augumentation index standardized for 75 beats per minute, *cSBP* central systolic blood pressure, *cDBP* central diastolic blood pressure, *oSBP* office systolic blood pressure, *oDBP* office diastolic blood pressure

^a^ The large decrease in the cSBP and oSBP as well as STD which is large might be due to three patients who experienced a high cSBP reduction as follows: 126–112, 133–116 and 146–124 mmHg while for the same patients the oSBP were: 132–111, 144–133 and 154–123 mmH

**Fig. 1:**
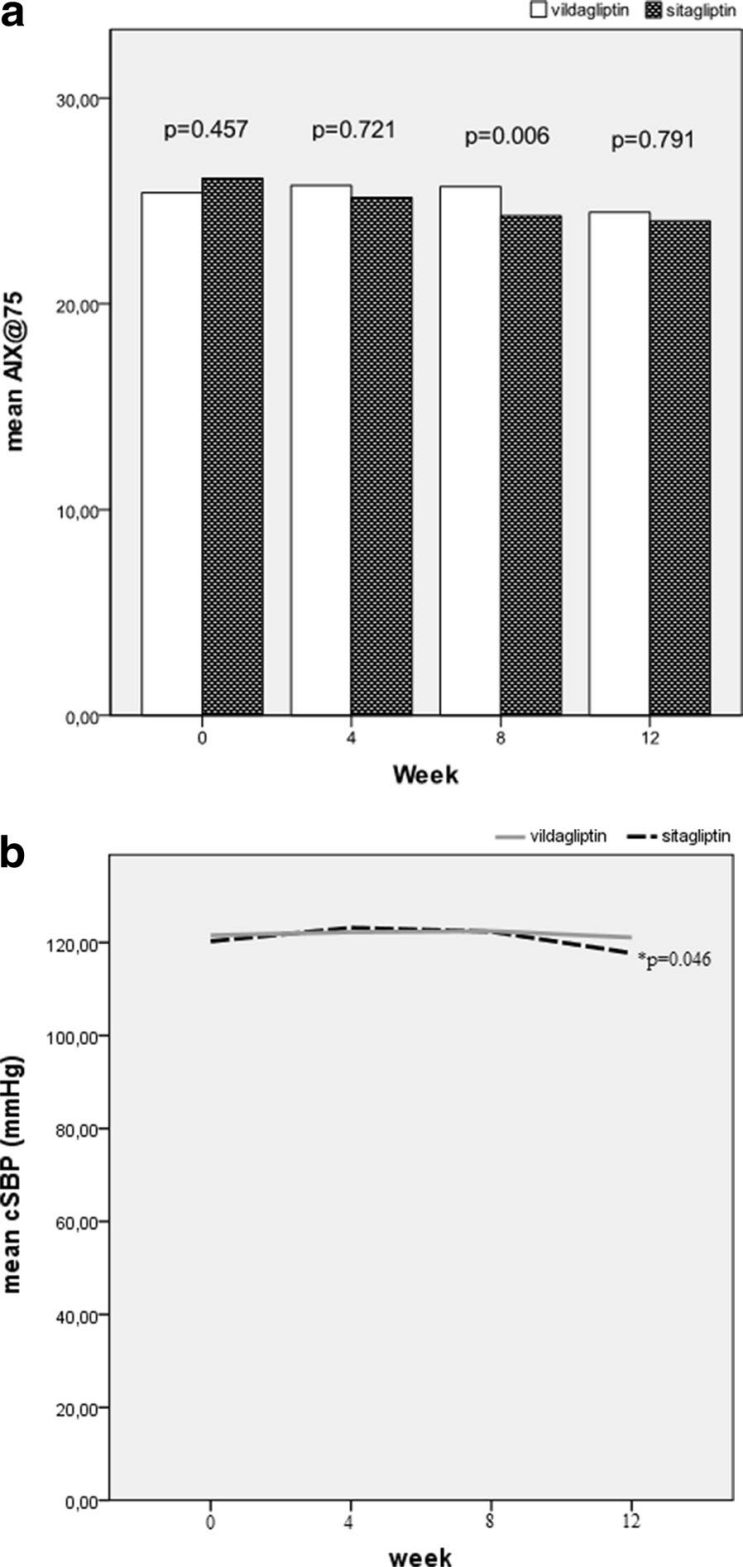
**a** Difference between sitagliptin and vildagliptin induced change in mean cAiX@75, **b** Difference between sitagliptin and vildagliptin induced change in mean cSBP

To evaluate the pharmacological action of DPP-4 inhibitors more precisely, we analyzed the results of DPP-4 treated cohort by stratifying them to sitagliptin and vildagliptin treated group. Sitagliptin resulted in significant oSBP [136.2 (12.3) vs 131.7 (10.5) mmHg, p = 0.037] and oDBP [84.8 (6.8) vs 74.1 (9.8) mmHg, p = 0.001] reduction while vildagliptin reached the significance only regarding oDBP [82.3 (10.1) vs 77.2 (10.4) mmHg, p = 0.007]. cDBP was also significantly reduced only for sitagliptin group (78.8 (9.6) vs 73.8 (9.6) mmHg, p = 0.002). In addition, hsCRP reduction was significant only for the sitagliptin group and was superior to vildagliptin [−0.84 (0.31) vs −0.31 (0.03) mg/L, p = 0.017]. Sitagliptin was superior to vildagliptin in cAiX@75 reduction in the 8th week of the study (25.3 vs 26.4, p = 0.006), however, the difference was lost by the study end (Fig. 1a). Finally, at the 12th week sitagliptin treated group had significantly lower cSBP compared to vildagliptin group (117.8 vs 122.4, p = 0.046) (Fig. 1b) although the reduction was not significant when compared to the cSBP value at the study beginning for neither gliptin.

## Discussion

The major finding of our study is that the 12-week treatment with DPP-4 inhibitors reduces the AS, PWV and 24-h SBP in T2DM patients. Furthermore, we showed that sitagliptin results in AS reduction earlier in the treatment period when compared to vildagliptin which might be due to their pharmacokinetic and pharmacodynamics properties [25]. However, the use of DPP-4 i did not result in mean HbA1c change during the study period.

There is accumulating evidence on global increase in the rates of T2DM associated cardiovascular events [26]. Obesity related inflammatory mediators, such as hsCRP are increased in diabetic state so inflammation related oxidative stress along with dyslipidemia seems to play a key role in the pathogenesis of vascular dysfunction [27–30]. In addition, increased AS, reduced aortic dispensability was shown in diabetic compared to nondiabetic population [31, 32]. Several studies reported AS as a strong predictor of cardiovascular morbidity and all-cause mortality in different populations and moreover, an independent predictor of 10-year mortality in patients with diabetes [21, 22].

Besides glycemic control, incretin-based treatment strategies for diabetes have focused on the reduction of CVD and its complications. There is a growing body of evidence suggesting that GLP-1-mimetics therapies have antiatherosclerotic and anti remodeling properties [11, 12]. However, if GLP-1 increase by DPP-4 inhibition exhibits the same vascular profile remains to be established.

It was recently shown that 6 weeks treatment with saxagliptin tends to improve central hemodynamics in T2DM patients [33]. Vildagliptin was reported to to improve AS in poorly regulated T2DM patients [34, 35] which is partially in accordance with our results. In a study by Mistry et al. [36] sitagliptin produced small but statistically significant reductions of 2–3 mmHg systolic and 1.6–1.8 mmHg diastolic BP measurements acutely (day 1) and at steady state (day 5) in nondiabetic patients with mild to moderate hypertension. Similar was reported recently by Marney et al. [37] in patients suffering from metabolic syndrome patients during placebo and low-dose ACE inhibition (5 mgenalapril), sitagliptin lowered blood pressure. These results are highly supported by experimental animals studies: AS, as assessed by PWV, was significantly increased in Western Diet fed mice (16 % increase) and was markedly decreased by DPP-4 inhibition [38].

We showed that both sitagliptin and vildagliptin treatment results in a significant hsCRP concentration reduction which is in accordance with the study data of Satoh-Asahara et al. [39] who were the first to report that sitagliptin treatment results in hsCRP reduction which was later confirmed for vildagliptin as well by Zografou et al. [35].

The addition of DPP-4 inhibitors to metformin (and gliclazide) also resulted in moderate waist circumference reduction. This is in agreement with previous studies and explained by the fact that although unlike mimetics, which boost GLP-1 activity leading to body weight reduction, DPP-4 inhibition works by slowing incretins degradation, therefore the level of circulating GLP-1 in inhibition therapy is significantly increased [40]. Furthermore, Koren et al. [17] recently reported a significant triglyceride concentration decrease in T2DM patients by 0.2 ± 0.5 mmol/L following sitagliptin treatment, while the change in the total serum cholesterol, LDL- and HDL-cholesterol did not reach the statistical significance. We also demonstrated a significant drop in triglyceride concentration, but in addition, we showed a significant LDL- and total serum cholesterol reduction which is in accordance with the data from the most recent meta-analysis discussing the DPP-4i on lipid profile [41]. Improvement of this common pattern of dyslipidemia in T2DM indicates that it might be a treatable risk factor for subsequent CVD.

Many pathophysiological mechanisms might explain these results. First, DPP-4 inhibition certainly acts partially throw increasing incretin levels (GLP-1) and the recent meta-analysis definitely indicate their cardiac friendly lipid profile [42]. Previous animal studies have shown that GLP-1 can induce a NO-dependent or -independent relaxation of arteries [43, 44]. Second, the increased DPP-4 activity was reported in T2DM when accompanied with a significant degree of insulin resistance (IR) [43], i.e., DPP-4 was recently proposed as a novel adipokine linking obesity to metabolic syndrome (MS) [45–47]. It should be kept in mind that IR is often associated with abnormal lipid metabolism and hypertension, and those two traditional CV risk factors may explain the increase in AS in T2DM and MS as well as the potential benefit of DPP-4 inhibition derived therapies.

## Conclusion

DPP-4 inhibitors could have a beneficial effect not only on blood glucose levels but also on blood pressure and artery function. It should be noted however that there are several limitations in our study. The greatest drawback of the study is the lack of placebo-treated group which emphasises the possibility that the DPP-4i observed therapy benefit might be due to lifestyle changes during the treatment period. In addition, due to small number of participants and 12-week follow up accompanied by the fact the study was uncontrolled and open-label, our results should be interpreted with caution. Further large randomized placebo controlled long-term studies are required to evaluate the effect of gliptines on vascular function, inflammation and dyslipidemia in patients with T2DM.
